# Current Trends in Orthognathic Surgery

**DOI:** 10.3390/medicina59122100

**Published:** 2023-11-30

**Authors:** Domenick Zammit, Russell E. Ettinger, Paymon Sanati-Mehrizy, Srinivas M. Susarla

**Affiliations:** 1 Department of Pediatric Surgery, Division of Plastic Surgery, McGill University Health Center, Montreal Children’s Hospital, Montreal, QC H3Z 1X3, Canada; dino.zammit@mcgill.ca; 2 Department of Surgery, Division of Plastic Surgery, University of Washington, Seattle, WA 98105, USA; 3 Craniofacial Center, Seattle Children’s Hospital, Seattle, WA 98105, USA; 4 Department of Plastic Surgery, University of Texas Southwestern Medical Center, Dallas, TX 75390, USA; paymon.sanati-mehrizy@utsouthwestern.edu; 5 Department of Oral and Maxillofacial Surgery, University of Washington, Seattle, WA 98195, USA

**Keywords:** orthognathic surgery, osteotomies, aesthetic problems

## Abstract

Orthognathic surgery has evolved significantly over the past century. Osteotomies of the midface and mandible are contemporaneously used to perform independent or coordinated movements to address functional and aesthetic problems. Specific advances in the past twenty years include increasing fidelity with computer-assisted planning, the use of patient-specific fixation, expanding indications for management of upper airway obstruction, and shifts in orthodontic-surgical paradigms. This review article serves to highlight the contemporary practice of orthognathic surgery.

## 1. Introduction

Orthognathic surgery has been a mainstay of craniomaxillofacial surgical treatment for over a century [1,2,3]. Osteotomies of the midface and mandible are utilized in contemporary craniomaxillofacial practice to address three-dimensional dysmorphology of the maxillomandibular complex, with positive effects on the occlusion, facial aesthetics, and management of airway obstruction.

**Figure 1:**
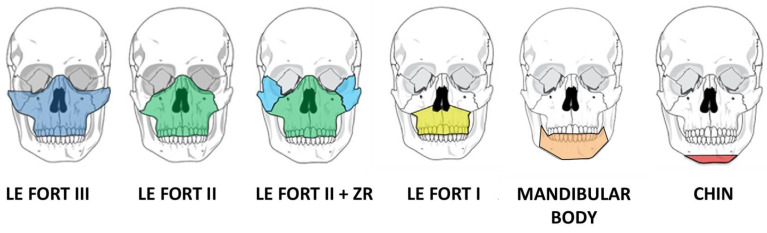
Osteotomies for moving the midface and mandible to address dentofacial or craniofacial differences.

**Figure 2:**
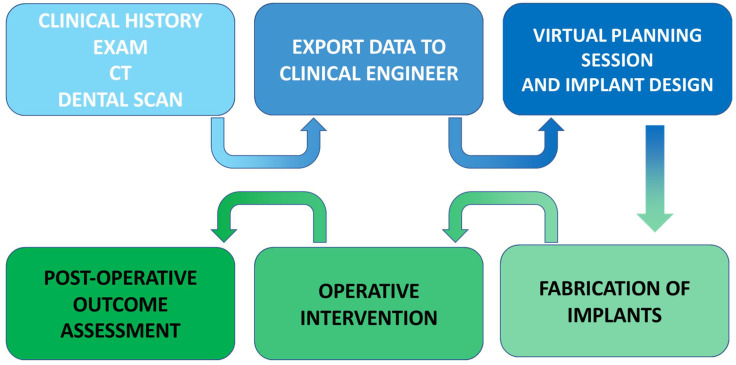
Contemporary workflow for computer-assisted surgical planning.

The development of orthognathic surgery has many parallels with the management of traumatic facial injuries. The patterns of midface fractures, initially described by Rene Le Fort and bearing his name, were translated into elective osteotomies of the facial skeleton in the 1950s–1970s through the pioneering work of numerous surgeons, including Norman Rowe, Paul Tessier, Hugo Obwegeser, and William Bell (Figure 1) [4,5,6,7,8]. With advances in anesthetic techniques as well as the advent of rigid fixation in the 1980s, operations became more predictable and routine. In the 1990s and the early part of the 21st century, iterative advancement of the facial skeleton via distraction osteogenesis added greatly to the armamentarium of techniques for surgeons to address congenital and developmental facial skeletal differences [9,10,11,12]. Advances in digital imaging and analysis allowed for increased understanding of the three-dimensional movements of the jaws and creation of surgical plans in a virtual environment, enabling surgeons to navigate complex anatomy and improve the interventions [13,14,15,16,17,18,19]. Computer-assisted surgical planning subsequently expanded to include computer-assisted design (CAD) and computer aided manufacturing (CAM) of patient-specific cutting guides and implants, enhancing the safety and efficacy of these procedures, allowing for increased efficiency for straightforward interventions and increased versatility for more complex interventions (Figure 2) [20,21,22].

**Figure 3:**
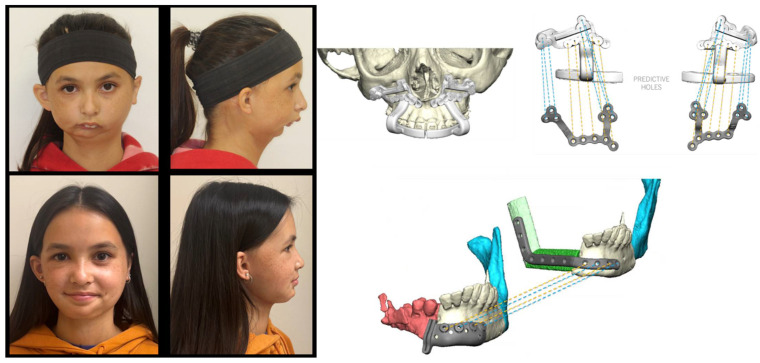
Complex reconstruction utilizing customized three-dimensional planning to address severe facial asymmetry and micrognathia via Le Fort I osteotomy, left mandibular sagittal split osteotomy, and two-segment free fibula flap.

Contemporary advances in orthognathic surgery include further advances in surgical planning, changes in coordinated orthodontic-surgical protocols, advanced distraction techniques, greater understanding of the changes in airway dynamics that accompany selected movements of the facial skeleton and expanding indications for patients with complex facial differences requiring simultaneous orthognathic surgery and free tissue transfer (Figure 3).

## 2. Advances in Surgical Planning

Classical surgical planning in orthognathic surgery required the use of cast dental models mounted on a semi-adjustable articulator to reproduce the relationship of the jaws to the cranial base. This process involved numerous steps with a high potential for accumulated errors and was time intensive (Figure 2. Planning largely focused on two-dimensional changes, specifically sagittal (front-back) and vertical (up-down) positioning of the jaws relative to each other and the cranial base. More specifically, historical planning was based largely upon the occlusion alone, rather than a comprehensive understanding of the changes in bone position and interferences that accompany changes in the dentate jaws (i.e., pitch, yaw, and roll). Higher fidelity images capture modalities and a greater understanding of the complexities of movements as rendered in a three-dimensional virtual surgical environment have allowed surgeons to more effectively and precisely address myriad skeletal deformities, with decreased operating time and hospital stays [22,23,24,25].

## 3. Surgery First/Surgery Only/Clear Aligner Therapy

**Figure 4:**
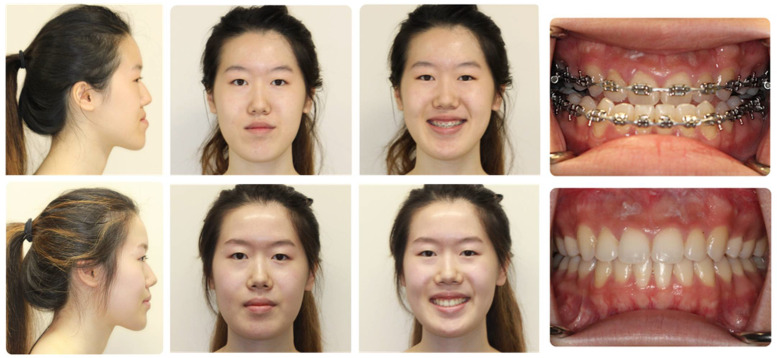
Traditional orthognathic surgical planning includes fixed orthodontic appliances for orthodontic decompensation, followed by surgical intervention, and a period of post-surgical orthodontic coordination. This patient with a class III malocclusion was treated with fixed appliances for 24 months prior to surgery (Le Fort I osteotomy and mandibular sagittal split osteotomies), followed by 6 months of post-surgical orthodontic care after surgery (total treatment time: 30 months).

Traditionally (Figure 4), orthognathic surgical treatment involves a sequential approach with presurgical orthodontic preparation (12–18 months), followed by surgery, and post-surgical orthodontic coordination (6–12 months) [26,27,28]. The pre-surgical stage is designed to “decompensate” the dentition such that the dental deformity matches the skeletal deformity. This is followed by surgical care where the bone position is altered to morphometric norms, and post-surgical orthodontic coordination in order to finish the occlusion to have optimal interdental contacts. Despite the conventional approach standing the test of time, drawbacks include prolonged treatment time as well as potential aggravation of facial aesthetics during the presurgical treatment period [26,27]. Recent advancements in the mechanics of tooth movements, stability of specific skeletal movements in the context of rigid fixation and understanding of the effects of surgery on tooth movement have allowed for the introduction of several different approaches to address skeletal dysplasia, including surgery-first and surgery-only approaches, as well as the use of clear orthodontic aligners in lieu of conventional metal braces. In appropriately selected patients, these protocols each offer several benefits, including reduced treatment time, greater quality of life, and improved patient satisfaction [26,27,28,29].

### 3.1. Conventional Orthognathic Surgical Paradigm

In patients with skeletal discrepancies, teeth naturally compensate to allow for a functional occlusion [27,28]. As a result, the inclination and position of the teeth are not directly proportional to the degree of the dentofacial deformity [27]. Traditional orthognathic surgical planning involves a period of presurgical orthodontic treatment to synchronize the dental deformity with the skeletal deformity by “decompensating” the teeth. This allows for a planned final occlusion wherein balanced forces are transmitted across the teeth, based upon their location within the arch, and the teeth are centered within the correct position in the alveolar housing [30]. It is not uncommon for patients to complain, as well as clinicians to warn, that the “bite was/is made worse” in preparation for orthognathic surgery. Once the dental deformity matches the skeletal deformity, surgical intervention is performed, followed by a period of post-surgical coordination to further align the teeth. Thus, total treatment times (beginning of pre-surgical orthodontics to end of post-surgical orthodontics) may be prolonged, with a mean of up to 36 months [28,29].

### 3.2. Surgery First Protocol (Figure 5)

In contrast to the conventional paradigm, “surgery-first” orthognathic treatment plans involve moving the jaws to the appropriate position with no or very limited pre-surgical orthodontic coordination [26,27,28,29]. The success of the surgery-first orthognathic approach lies in its utilization of the body’s natural compensatory adaptation process, eliminating the need for a decompression procedure. By applying only postsurgical orthodontics, the surgery-first approach takes advantage of the regional accelerated phenomenon (RAP), a known facilitator of postsurgical dental movement. In essence, RAP is a reaction of the soft and hard tissues to noxious stimuli, which directly leads to increased healing capacities of the tissues. Previous studies have shown that there is at least a twofold acceleration in post-operative orthodontic tooth alignment, therefore allowing post-surgical orthodontics in a surgery first candidate to catch up the non-existent pre-surgical orthodontic treatment time [27]. This allows for utilization of RAP to maximize the effectiveness of postsurgical orthodontic treatment in achieving desired dental movements. By implementing the surgery-first approach, the focus is shifted towards achieving the desired dental movements following surgery, rather than extensively preparing the dentition before any surgical procedure.

A standardized workflow for surgery-first orthognathic has been extensively described by Choi et al. wherein a dental model is used for simulation surgery prior to the actual surgical procedure [26,31]. This simulation aims to create an appropriate splint in order to estimate the required postsurgical orthodontic treatment required:

1. Preoperative Evaluation: Initially, the patient’s occlusion is assessed through the standard model mounting process. This evaluation provided a baseline understanding of the existing dental and skeletal relationships.
2. Model Setup: The teeth that are already adapted to the skeletal discrepancy are simulated and reorganized into their predicted locations. Each tooth is individually analyzed, simulated, and separated, mimicking presurgical orthodontic treatment. This step allows for a comprehensive assessment of tooth movements necessary for achieving optimal occlusion.
3. Simulation of Orthognathic Surgery: The next phase involves simulating orthognathic surgical movements on the model, similar to the standard approach. The simulated surgical movements demonstrate the potential occlusal outcome after presurgical orthodontics and orthognathic surgery on the model.
4. Surgical temporary occlusion: The teeth are restored to their original positions before any presurgical orthodontic treatment on the dental model. By using the original teeth model, can accurately replicate the dental condition that would reflect the outcome of orthognathic surgery without presurgical orthodontics.
  - Once the teeth are repositioned to their pre-orthodontic treatment state, one can create intermediate and final splints for orthognathic surgery without presurgical orthodontics.
  - The creation of these intermediate and final splints is based on the results of the simulated model surgery.
  - By incorporating the surgical plan derived from the simulation, splints that are aligned with the desired surgical outcome can be fabricated.

Ebkar et al. recently explained their method of surgery first approach using computer-assisted surgical planning (CASP) [32]. In brief, they simulate both the surgical as well as orthodontic movements in a virtual 3D model. While this novel utilization of CASP has a potential role as we become more familiar with surgery first treatment, it should be noted that after the planning sessions, patients were required to wear passive maxillary/mandibular splints to prevent any further teeth movements/rotations/inclinations [32].

**Figure 5:**
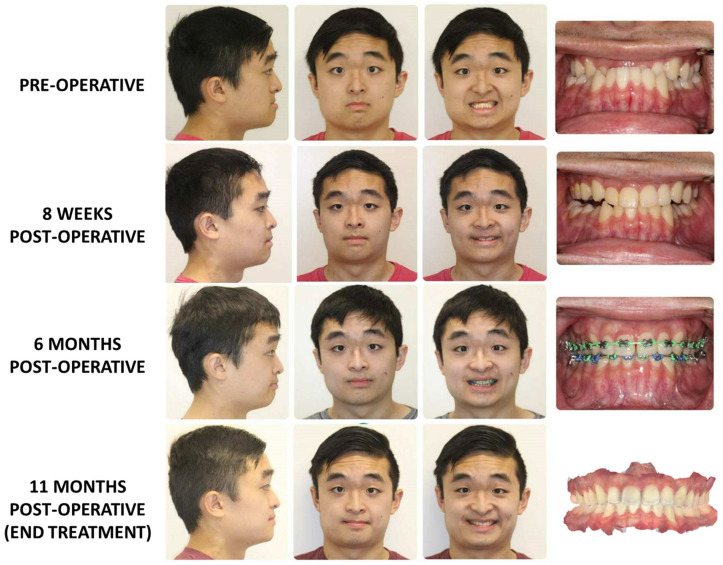
This patient underwent treatment for a class III skeletal malocclusion using a “surgery-first” approach. A Le Fort I osteotomy was performed without pre-surgical orthodontic coordination. Fixed appliances were placed at 8 weeks post-operatively, with rapid improvement noted at 6 months post-operatively. The appliances were removed at 11 months post-operatively. Total treatment time was approximately 1 year.

As one might conclude, the initial preparation for a surgery-first case requires a sophisticated analysis of the current state and thoughtful projection of the future state of both the occlusion and facial form. This additional effort does pay significant dividends however, as many studies have shown that the surgery-first approach may have an overall shorter treatment time (reduced by 8 months relative to conventional treatment) [28]. Furthermore, studies have shown that surgery first is more efficacious, with similar stability and surgical outcomes compared to the traditional approach [29]. As physicians become more aware and comfortable with the concept of surgery-first approach for the correct patient, the field will continue to evolve as we become more innovative in our approach to orthognathic surgery. While the most common contemporary indication is in the treatment of class III patients (Figure 5), there are expanding indications for the surgery-first approach, including patients with craniofacial differences, class II malocclusuions, and asymmetries [28,29,30,31,32,33,34]. One such pathology that has been more recently looked at is its role in the treatment of facial asymmetry cases. A recent study by Choi et al. has demonstrated through the use of AI assessing cephalometric measurements that relapse rates are similar in both orthodontics first and surgery first approaches [33]. Moreover, they have been able to show that despite the difficulties in both the assessment and treatment of patients with facial asymmetries, using the surgery first model, they can achieve predictable results [31].

Overall, the surgery-first orthognathic approach offers a transformative alternative to traditional orthodontic treatments, improving treatment efficiency, patient satisfaction, and postoperative outcomes. As research continues and new paradigms emerge, we anticipate further advancements in the field, broadening the scope of the surgery-first approach and enhancing its applicability in various clinical scenarios.

### 3.3. Surgery-Only Protocol (Figure 6)

In patients without the need for significant alterations in the occlusion, but the need for the reorientation of the jaw position to address asymmetry or for primary aesthetic purposes, a “surgery-only” protocol can be utilized. This approach is not novel, per se, as moving the jaws without altering the occlusion has been practiced for decades and shares similarities with maxillofacial trauma surgery. What has changed has been the ability of the surgeon to utilize customized three-dimensional planning to effectively move the jaws reliably and consistently without the use of intermaxillary fixation appliances peri-operatively. The use of customized cutting guides and fixation plates reduces the need to rely on occlusal splints, in many instances obviating the need for splints altogether [35]. Asymmetry corrections in particular benefit from these types of devices, as the precise placement of the fixation device recapitulates the planned skeletal movement created in the virtual environment.

**Figure 6:**
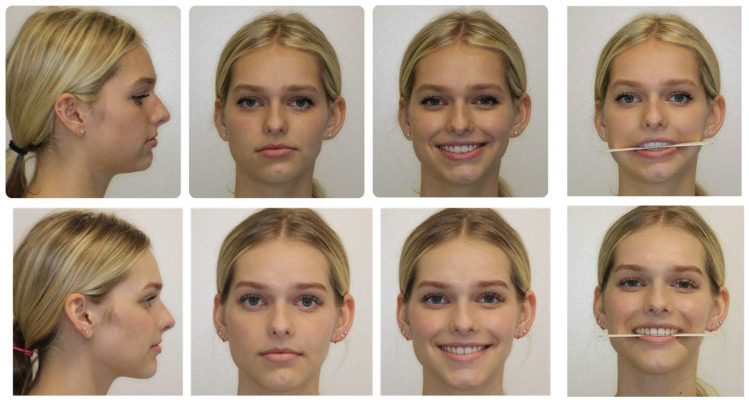
This patient was noted to have a facial symmetry related to left-mandibular ramus-condyle unit hypoplasia but had undergone prior orthodontic correction in early adolescence. Surgical intervention via Le Fort I osteotomy, bilateral sagittal split osteotomies, and genioplasty was undertaken to address the skeletal asymmetry and retrusion without altering the occlusion.

### 3.4. Clear Aligner Therapy (Figure 7)

The use of clear aligners has allowed for patients who require some degree of orthodontic coordination to avoid the challenges associated with fixed metallic braces, including hygiene, aesthetic concerns, and patient comfort [35,36,37,38,39]. As the indications for clear aligner therapy have expanded, many orthodontists have transitioned to offering this approach for patients with skeletal malocclusions requiring a coordinated orthodontic-surgical approach for management.

Clear aligner therapy in orthognathic surgery requires a series of removable appliances worn for 20–22 h per day [37,38,39]. These devices allow for iterative tooth movement toward a planned occlusion and have been effectively used in isolation for orthodontic treatment of mild to moderate malocclusions and have recently gained popularity for skeletal malocclusions requiring orthognathic surgery. As with conventional treatment, a successful surgical result requires close coordination between the orthodontist and surgeon. Once the treatment plan has been developed, the patient wears a series of active aligners that move the teeth into the planned pre-surgical position. Once this position has been achieved, passive trays are utilized to prevent tooth movement during the perioperative period. One to two weeks after initiation of passive aligner treatment (to allow for some settling of the teeth when transitioning from active to passive systems), pre-surgical computer-assisted planning is completed, similar to the conventional approach. Surgical intervention is completed in the standard fashion, using temporary intermaxillary fixation devices or splint less surgery with customized cutting guides and patient-specific implants. Post-surgical passive aligners are used for 4–6 weeks after surgery, then the patient resumes active orthodontic treatment with post-surgical aligners.

The use of clear aligners in orthognathic surgery has preliminarily showed promise, with several studies suggesting comparably efficacy to fixed appliance therapy, with the added benefits of increased patient acceptance, decreased swelling, and, perhaps, shorter treatment times [35,36,37,38,39].

**Figure 7:**
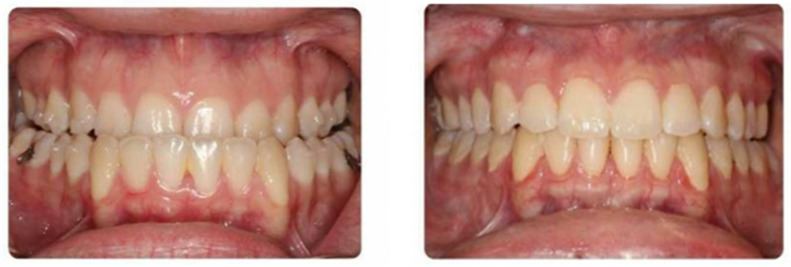
Correction of a class III skeletal malocclusion with clear aligner therapy and orthognathic surgery. Pre-treatment occlusion (top) and end-treatment occlusion (bottom).

## 4. Orthognathic Surgery for Obstructive Sleep Apnea

Obstructive sleep apnea (OSA) is a common disorder that affects about 1/3 of the adult population but can be seen in all age groups [40,41,42,43,44,45]. It is characterized by repeated episodes of partial or complete obstruction of the upper airway during sleep, leading to disrupted sleep and decreased oxygen levels in the body. The most prevalent patient complaints typically involve daytime sleepiness and snoring. From a clinical standpoint, this disrupted sleep pattern leads to a compromised cycle characterized by apneas and hypopneas. Apneas are defined as a cessation of airflow lasting at least 10 s, while hypopneas occur when there is a diminished respiratory drive leading to desaturation that resolves through arousal [40,41]. Over time, this chronic hypoxic state creates an unfavorable physiological environment, potentially causing detrimental effects on various organ systems [45,46]. Many studies have shown that OSA can be the contributing factors to such diseases as hypertension, congestive heart failure, and cerebrovascular disease [40,41,42].

### 4.1. Work-Up and Measures of Severity

**Figure 8:**
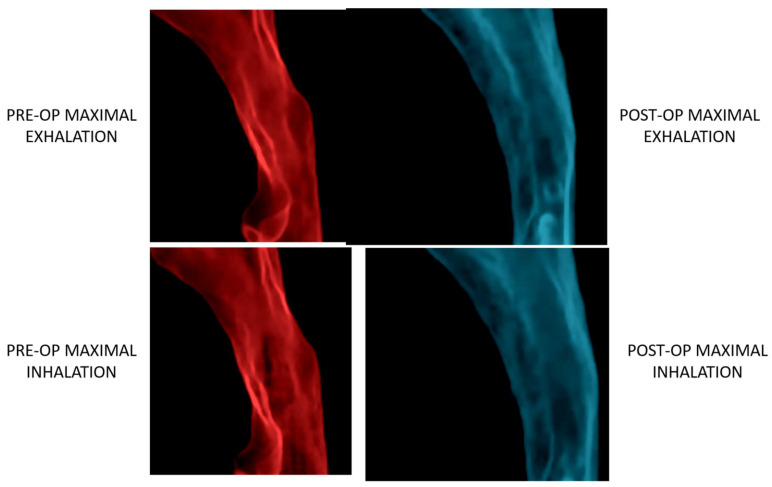
4D images showing airway changes pre-operatively and 4 months post-operatively in a patient with bimaxillary retrusion undergoing maxillomandibular advancement and genioglossus advancement. The pre-operative images show a non-uniform airway shape with notable collapse during maximal inspiration. Post-operatively, the airway shape is more uniform, with notable volumetric expansion during all phases of the respiratory cycle.

Treatment of OSA beings with an appropriate history and physical exam, but ultimately requires diagnostic testing. One should ascertain symptoms of OSA as described above, and note any contributing factors on physical exam, such as obesity, micro-retrognathia, as well as a complete intra-oral exam. If experienced, one may also employ fiberoptic nasopharyngoscopy to look at all levels of the naso-oro-pharynx for other causes of airway obstruction [40,41,42,43,44,45]. These findings can then be classified based on the Fujita scale, with Type I being palatal obstruction, Type II as both palatal and tongue base obstruction, and type III occurring at the tongue base only. Enhanced by incorporating standard measurements from a lateral cephalogram, these measurements can offer insights into the anatomical factors contributing to the obstruction in patients with OSA. However, they do not provide a comprehensive assessment of the severity of OSA. In recent years, the utilization of MRI and CT scans has significantly improved the identification and characterization of restricted areas within the airways, enabling volumetric analysis as well [46,47,48,49,50,51,52]. An expanding frontier is the use of four-dimensional computed tomography assessment (imaging taken over one respiratory cycle) to obtain a more dynamic understanding of the airway (Figure 8) [49,51].

Currently, the gold standard diagnostic tool for OSA is the polysomnogram [40,41,43]. This can assess the cardiorespiratory system to give an indication on the body’s oxygenation status, and along with an electroencephalogram, electrooculogram, and electromyogram, estimates apneas, hypopneas, and respiratory related events during sleep. These will then give a measure of apnea-hypoxia index (AHI). As with many disease processes, OSA severity is graded on a scale depended on the AHI measurements: mild is an AHI between 5–15 with daytime sleepiness, moderate is an AHI between 15–30, and severe is an AHI > 30 [40,41,43]. It is using these measurements that we can get an accurate idea of whether or not the treatment modalities prescribed to the patient are having an effect.

### 4.2. Non-Invasive Treatment Options

Non-invasive treatment options for OSA include weight loss, sleep hygiene, various oral appliances, and continuous positive airway pressure (CPAP) or bilevel positive airway pressure (BiPAP). While there is plenty of evidence to support the use of these non-invasive treatment modalities in terms of improving both symptoms and AHI, they are not curative [40,41,42,43,44]. And while CPAP therapy is the gold standard for OSA treatment, some patients may not tolerate or adhere to CPAP therapy. In such cases, surgical options, such as orthognathic surgery, have emerged as attractive alternative treatment options [40,41,42,43,44,45,53,54].

### 4.3. Surgical Treatment Options

The contemporary view on surgical treatment for OSA revolves around a two-tiered approach for surgical management [40,41,42,43,44,45]. The goal of this system is to decreases the risks of surgery and develop an escalating approach to treating OSA. Initial Phase I treatment consists of procedures such as tonsillectomy/adenoidectomy, intranasal procedures (septoplasty, turbinectomy), uvolopalatopharyngoplasty, genioglossus advancement, and other site-specific interventions. Phase II involves either a maxilla-mandibular advancement (MMA), tongue reduction, or hypoglossal nerve stimulation [40,41,42,43,44,45]. It is important to note that some patients may need to go through both phases, and a patient must undergo a polysomnogram 4–6 months after Phase I to determine if they must move on to phase II treatment. Despite all the various options, MMA may be the most effective surgical procedure to address OSA in skeletally mature patients.

### 4.4. Orthognathic Surgery for the Treatment of OSA

**Figure 9:**
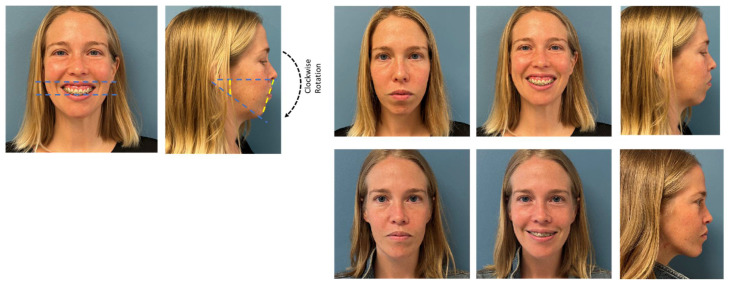
Bimaxillary retrusion with clockwise rotation of the maxillomandibular complex. Orthognathic surgery with maxillomandibular advancement and counterclockwise rotation can dramatically improve not only facial aesthetics, but also afford increased airway expansion for a given amount of linear sagittal movement.

As our understanding of the underlying pathological mechanisms behind OSA continue to evolve, CMF surgeons have utilized their armamentarium of surgical techniques to successfully employ orthognathic surgery as a powerful tool for the treatment of OSA [40,41,42,43,44,45,46,53,54,55,56,57]. Specifically, anomalies such as micro/retrognathia, transverse maxillary deficiency, bimaxillary retrusion, and a steep occlusal plane are all orthognathic factors that can contribute to OSA. Recent studies and meta-analyses have shown modification of traditional orthognathic techniques can improved both patient outcomes and satisfaction [55,57,58,59,60]. In general, orthognathic surgery involves repositioning the facial skeleton to correct any facial asymmetries or dentofacial discrepancies but can also be employed to enlarge the upper airway and improve breathing during sleep. Despite being invasive, MMA is currently the most performed orthognathic procedure for OSA. Contemporary MMA involves advancing the upper and lower jaws forward to enlarge the airway and improve breathing. Some reports in the literature suggest that a total maxillary–mandibular complex movement of at least 10 mm is required to address OSA [40,41,42,43,44,45]. Additionally, recent advances have shown how incorporation of a counterclockwise (CCW) rotation into MMA can improve patient outcomes even further [40,41,42,43,44,45,56,61]. This is achieved by improving the occlusal plane angle on pharynx morphology, further mandibular advancement, which ultimately translate to improved polysomnography results. The main advantage of incorporating CCW rotation is in patients wherein too much of a bimaxillary advancement would compromise facial aesthetics [40,41,42,43,44,45,62]. This approach stems from the fact that each degree of CCW rotation corresponds to an advancement of 0.71 mm without requiring additional, or minimal, advancement of the maxillo–mandibular complex [56]. Therefore, a 10-degree change in occlusal plane angle would lead to a mandibular advancement of 7.1 mm. The linear advancement that would then be left to achieve the 10 mm needed would only be 3 mms. As one can imagine, the facial aesthetics would differ immensely between a 3 mm complex advancement versus a 10 mm advancement. This finding recapitulates observations regarding the correlation of sagittal midface movements in sub-cranial surgery as a function of counterclockwise palatal plane rotation [54]. Facial morphologic changes seen in a patient undergoing bimaxillary advancement with counterclockwise rotation via Le Fort I osteotomy and bilateral mandibular sagittal split osteotomies are shown in Figure 9.

Evidence on the successful outcomes stems from various meta-analyses looking at measures of AHI pre and post-surgery, with a success rate of up to 80% [55]. However, it is important to note that the lower the initial AHI/BMI, the higher the chance of success of reducing AHI to normal levels [55]. Despite this caveat, most patients undergoing MMA report improved symptoms, with those having the highest pre-operative AHI noting the greatest degree of improvement. While there is no definitive consensus in the literature regarding the thresholds for acceptable candidates for surgery, most centers utilize a combination of demographic, health status, anatomic, and cephalometric parameters to determine whether the benefits of the procedure outweigh the risks [40,41,42,43,44,45,55].

As more surgeons begin to practice MMA as a means for OSA treatment, experts are rapidly developing various pre, peri and post operative protocols to ensure increased patient safety. An example of a commonly used protocol is that proposed by Barrerra et al. [40].

1. Reduction of intraoperative airway risks using fiberoptic intubation to appropriately assess the airway in the presence of operating surgeon.
2. Plan for extubation at the end of the procedure, prior to ICU transfer.
3. ICU admission and aggressive hypertension management in the acute post-operative period
4. Pre- and post-operative use of nasal CPAP for 2 weeks prior to surgery if respiratory disturbance index is greater than 40 and a low oxyhemoglobin desaturation less than 80.
5. Use of humidified oxygen at 35% through face tent if do not meet requirements for nasal CPAP.
6. Continuous oximetry until discharge
7. Judicious use of IV narcotics: Nurses must monitor patients after the use of any IV narcotics, as well as no patient should get a patient-controlled anesthesia pump.
8. Discharge criteria involved adequate oral intake, pain control with oral medication, documented reduction of facial edema, ability to tolerate nasal CPAP if indicated.

In summary, orthognathic surgery has emerged as an effective treatment option for OSA patients who cannot tolerate or adhere to CPAP therapy. Recent advances in orthognathic surgery have led to improved outcomes and increased patient satisfaction. These advances include improvements in surgical techniques, patient selection algorithms, and understanding of the physiology of the upper airway in OSA. Further research is needed to continue improving orthognathic surgery for OSA and expanding its application to a broader patient population.

## 5. In-House Computer-Assisted Surgical Planning

With the advent of CASP, orthognathic surgery has become more precise in execution and far less cumbersome in surgical planning [13,14,15,16,17,18,19,20,21]. Rather than classically performing model surgery on plaster molds and a facebow registration, the modern orthognathic surgeon can generate a digital three-dimensional skull model on which virtual surgery can be performed, allowing for real-time analysis and visualization of these anticipated movements. Ultimately, CASP allows for 3D printed surgical splints, which allow for the execution of a precise plan in the operating room.

However, a number of limitations exist within the traditional CASP model. The existing software to perform model surgery can be complex, requiring a trained engineer to import imaging data and manipulate the simulated skeleton. As such, this requires a web conference for collaboration between engineers, surgeons, and local representatives, which can be challenging to coordinate. Furthermore, due to the demand for these services, the time from CASP session to receiving the surgical splints can be prohibitive. Finally, there is a potentially substantial cost from third-party vendors for the use of proprietary platforms for planning, personnel, and material fabrication. To the extent that these interventions reduced operative times (as suggested by multiple studies), they can be cost-effective. However, surgeons across multiple centers have begun to perform in-house CASP and splint printing for orthognathic surgery using commercially available software and 3-dimensional printers, to potentially reduce costs specifically related to outsourcing the planning modality.

While the existing literature on this topic is fairly limited, initial reports are promising. As reported by De. Rui et al., three-dimensional printed surgical guides planned and printed in-house demonstrated high rates of accuracy for the majority of measured anatomic variables [63]. Additionally, an article published in 2021 by Mascarehas et al. reports on the cost-effective nature of in-house CASP and surgical splint printing for orthognathic surgery. Based on their analysis of 35 consecutive patients undergoing single jaw surgery, the total time needed from initial scanning to splint manufacturing took 5–9 h and with substantially reduced direct costs [64]. The authors remark, however, that the initial investment can be quite steep, as an institution would be required to purchase a high-power computer, intra-oral or model scanner, a 3D printer, and printing material. However, once this initial investment is complete, each generated splint will be available much sooner and at a lower cost than those generated by the commercial planning companies. Additionally, in-house software platforms for digital analysis, including the use of automated, artificial intelligence processes, are showing promise for advancing the efficiency and accuracy of diagnosis and treatment of dentofacial deformities [65,66,67]. Finally, with increasing understanding of digital image analysis and three-dimensional planning, it is possible that the computerized surgical planning process may be largely transferred to in-house engineers or even become fully automated.

**Figure 10:**
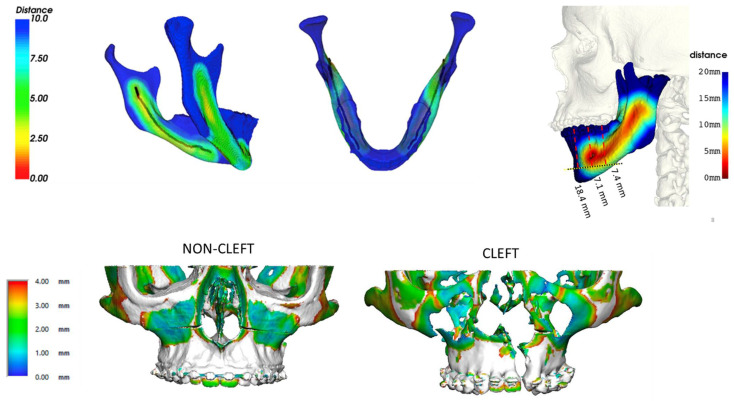
In-house automated image analysis is an evolving area that can help improve our understanding of the array of diagnoses that accompany skeletal malocclusions. While treatment planning will always be patient-specific, understanding of the global differences between diagnosis can have important implications for how we approach treatment planning. For example, the path of the inferior alveolar nerve and its proximity to the sagittal split osteotomy cut (top, left) and extended genioplasty (top, right) as well as variability in bone thickness in the midface (bottom) between cleft and non-cleft maxillae. Reprinted, with permission, from reference [67].

In-house high-fidelity imaging also affords centers an opportunity to develop automated protocols for evaluating regional anatomy. This can be useful for generating normative data to better understand normal and abnormal anatomy and develop practical models for teaching and precise execution of osteotomies (Figure 10).

## 6. Summary

There has been tremendous innovation in orthognathic surgery over the past century, with a particularly rapid evolution over the past twenty years. Advances in imaging, computer-aided surgical planning, patients-specific fixation, and improved understanding of the pathophysiology of airway obstruction have allowed surgeons to treat an ever-expanding group of diagnoses and effectively address even the most complex anatomic presentations. Further evolution along this trajectory may be directed toward evaluating dynamic airway changes following jaw advancement, correlations between soft tissue and bony changes, and refined methods for reducing orthodontic and surgical treatment time. As the field continues to evolve, further research and advancements are necessary to improve surgical techniques, patient selection algorithms, and broaden the application of orthognathic surgery to a wider patient population.

## Data Availability

This study did not report any data.
